# Multidrug-resistant tuberculosis

**DOI:** 10.1186/1471-2334-8-10

**Published:** 2008-01-25

**Authors:** Ellen M Zager, Ruth McNerney

**Affiliations:** 1 London School of Hygiene & Tropical Medicine, Keppel Street, London, WC1E 7HT, UK

## Abstract

**Background:**

With almost 9 million new cases each year, tuberculosis remains one of the most feared diseases on the planet. Led by the STOP-TB Partnership and WHO, recent efforts to combat the disease have made considerable progress in a number of countries. However, the emergence of mutated strains of *Mycobacterium tuberculosis *that are resistant to the major anti-tuberculosis drugs poses a deadly threat to control efforts. Multidrug-resistant tuberculosis (MDR-TB) has been reported in all regions of the world. More recently, extensively drug resistant-tuberculosis (XDR-TB) that is also resistant to second line drugs has emerged in a number of countries. To ensure that adequate resources are allocated to prevent the emergence and spread of drug resistance it is important to understand the scale of the problem. In this article we propose that current methods of describing the epidemiology of drug resistant tuberculosis are not adequate for this purpose and argue for the inclusion of population based statistics in global surveillance data.

**Discussion:**

Whereas the prevalence of tuberculosis is presented as the proportion of individuals within a defined population having disease, the prevalence of drug resistant tuberculosis is usually presented as the proportion of tuberculosis cases exhibiting resistance to anti-tuberculosis drugs. Global surveillance activities have identified countries in Eastern Europe, the former Soviet Union and regions of China as having a high proportion of MDR-TB cases and international commentary has focused primarily on the urgent need to improve control in these settings. Other regions, such as sub-Saharan Africa have been observed as having a low proportion of drug resistant cases. However, if one considers the incidence of new tuberculosis cases with drug resistant disease in terms of the population then countries of sub-Saharan Africa have amongst the highest rates of transmitted MDR-TB in the world. We propose that inclusion of population based statistics in global surveillance data is necessary to better inform debate on the control of drug resistant tuberculosis.

**Summary:**

Re-appraisal of global MDR-TB data to include population based statistics suggests that the problem of drug resistant tuberculosis in sub-Saharan Africa is more critical than previously perceived.

## Background

Control of tuberculosis (TB) remains one of the most serious challenges to global health. In 2005 there were an estimated 8.8 million new cases and 1.6 million deaths [1]. TB is predominantly a disease of poverty with over 80% of cases occurring in Asia or Africa. Although the greatest numbers of patients live in the highly populous countries of Asia the highest incidence of disease is found in the WHO region of Africa. Nine countries in sub-Saharan Africa have recently reported estimated annual incidences in excess of 600 cases per 100,000 [2], a burden of disease not witnessed since before the advent of chemotherapy. The continued rise of TB in this region may be largely attributed the AIDS pandemic combined with weak healthcare delivery systems.

A new and potentially devastating threat to TB control is the emergence of strains that cannot be cured by standard anti-tuberculosis drug regimens [3]. Drug resistant tuberculosis commonly arises through the selection of mutated strains by inadequate chemotherapy. Resistance to at least the two major anti-tuberculosis drugs, isoniazid and rifampicin has been termed multidrug-resistant tuberculosis (MDR-TB). Treatment of MDR-TB requires prolonged and expensive chemotherapy using second-line drugs of heightened toxicity. Should resistance to the second line drugs also arise then the disease becomes virtually untreatable. Extensively drug resistant-tuberculosis (XDR-TB) has been reported in all regions of the world [4]. XDR-TB is defined as resistance to at least rifampicin, isoniazid, a second line injectable drug (capreomycin, kanamycin or amikacin) and a fluoroquinolone [5]. Control of drug resistant tuberculosis requires a strong health infrastructure to ensure the delivery of effective therapy coupled with surveillance and monitoring activities to enable timely intervention to limit transmission and spread of the disease. It is paradoxical that drug resistance develops and flourishes in those very settings least able to deal with it. The recent report from KwaZulu Natal Province in South Africa of an outbreak of XDR-TB where rapid progression to death was observed in 98% of patients demonstrates the vulnerability of sub-Saharan Africa to outbreaks of untreatable disease [6].

Although studies demonstrating successful treatment outcomes for MDR-TB cases have been reported from a number of settings [7], the allocation of resources to detect and treat MDR-TB in poor resource settings remains controversial [8]. Whereas some advocate that priority be given to the effective treatment of drug sensitive disease, thus preventing the emergence of drug resistance [9], others argue that drug resistant cases should be detected and treated both for the good of the individual and to reduce ongoing transmission of drug resistant disease [10]. Inevitably, decisions on resource allocation are based on the perceived burden of disease. Data on the prevalence of drug resistant tuberculosis are currently presented as the proportion of cases found resistant to anti-tuberculosis drugs. This article discusses global data on multi-drug resistant tuberculosis and argues that reporting would be greatly improved by the inclusion of population based statistics. The proposition is illustrated by reference to sub-Saharan Africa which, while considered a low risk setting by traditional reporting methods, is shown here to have amongst the highest levels of transmitted MDR-TB in the world.

## Discussion

In 1994 WHO and the International Union Against Tuberculosis and Lung Diseases (IUATLD) established a Global Surveillance Project to standardise methodology, collect and analyse data on the extent of drug resistance and to monitor trends over time [11]. The project has reported the prevalence of drug resistance as the proportion of tuberculosis cases resistant to anti-tuberculosis drugs. A number of countries in Eastern Europe and the former Soviet Union and some regions of China have been identified as having a high prevalence of MDR-TB [12]. For those geographical settings in sub-Saharan Africa for which data is available the prevalence of MDR-TB is low. This has led to the suggestion that measures to control drug resistance in Africa should not be considered a high priority [9]. However, the very high incidence of tuberculosis in this region suggests that the number of MDR-TB cases within the population and the consequent risk of transmission may be significant. Resistance detected in previously untreated (new) cases provides an indication of transmission of drug resistant disease. To this end we have undertaken a re-analysis of available surveillance data to provide estimates of the incidence of MDR-TB in previously untreated cases per 100,000 of the population.

Drug resistance surveillance data published by the WHO and the International Union Against Tuberculosis and Lung Diseases Global Surveillance Project was used to estimate the incidence of previously untreated TB cases having multi-drug resistant disease. Data was sourced from the WHO/IUATLD Global Project on Anti-Tuberculosis Drug Resistance Surveillance reports of 2000 and 2004 [12,13]. The estimated incidence of tuberculosis for the year when the surveillance was carried out and the proportion of cases found MDR were used to calculate the incidence of MDR-TB per 100,000 of the population. Where necessary, population data was supplemented by reference to the US Census Bureau's International Data Base [14]. To allow estimation of the incidence of MDR-TB cases arising from transmission only data from previously untreated (new) cases were selected for analysis.

The following formulae may be used to estimate incidence of MDR-TB in the population:

Incidence of MDR-TB per 100,000 of the population=Number new cases of MDR-TB in the populationPopulation surveyed×100,000
 MathType@MTEF@5@5@+=feaagaart1ev2aaatCvAUfKttLearuWrP9MDH5MBPbIqV92AaeXatLxBI9gBaebbnrfifHhDYfgasaacPC6xNi=xI8qiVKYPFjYdHaVhbbf9v8qqaqFr0xc9vqFj0dXdbba91qpepeI8k8fiI+fsY=rqGqVepae9pg0db9vqaiVgFr0xfr=xfr=xc9adbaqaaeGacaGaaiaabeqaaeqabiWaaaGcbaGaeeysaKKaeeOBa4Maee4yamMaeeyAaKMaeeizaqMaeeyzauMaeeOBa4Maee4yamMaeeyzauMaeeiiaaIaee4Ba8MaeeOzayMaeeiiaaIaeeyta0KaeeiraqKaeeOuaiLaeeyla0IaeeivaqLaeeOqaiKaeeiiaaIaeeiCaaNaeeyzauMaeeOCaiNaeeiiaaIaeeymaeJaeeimaaJaeeimaaJaeeilaWIaeeimaaJaeeimaaJaeeimaaJaeeiiaaIaee4Ba8MaeeOzayMaeeiiaaIaeeiDaqNaeeiAaGMaeeyzauMaeeiiaaIaeeiCaaNaee4Ba8MaeeiCaaNaeeyDauNaeeiBaWMaeeyyaeMaeeiDaqNaeeyAaKMaee4Ba8MaeeOBa4Maeyypa0tcfa4aaSaaaeaacqqGobGtcqqG1bqDcqqGTbqBcqqGIbGycqqGLbqzcqqGYbGCcqqGGaaicqqGUbGBcqqGLbqzcqqG3bWDcqqGGaaicqqGJbWycqqGHbqycqqGZbWCcqqGLbqzcqqGZbWCcqqGGaaicqqGVbWBcqqGMbGzcqqGGaaicqqGnbqtcqqGebarcqqGsbGucqqGTaqlcqqGubavcqqGcbGqcqqGGaaicqqGPbqAcqqGUbGBcqqGGaaicqqG0baDcqqGObaAcqqGLbqzcqqGGaaicqqGWbaCcqqGVbWBcqqGWbaCcqqG1bqDcqqGSbaBcqqGHbqycqqG0baDcqqGPbqAcqqGVbWBcqqGUbGBaeaacqqGqbaucqqGVbWBcqqGWbaCcqqG1bqDcqqGSbaBcqqGHbqycqqG0baDcqqGPbqAcqqGVbWBcqqGUbGBcqqGGaaicqqGZbWCcqqG1bqDcqqGYbGCcqqG2bGDcqqGLbqzcqqG5bqEcqqGLbqzcqqGKbazaaGccqGHxdaTcqaIXaqmcqaIWaamcqaIWaamcqGGSaalcqaIWaamcqaIWaamcqaIWaamaaa@BF94@

Incidence of MDR-TB per 100,000 of the population=Proportion TB cases MDR100×Incidence of TB per 100,000
 MathType@MTEF@5@5@+=feaagaart1ev2aaatCvAUfKttLearuWrP9MDH5MBPbIqV92AaeXatLxBI9gBaebbnrfifHhDYfgasaacPC6xNi=xI8qiVKYPFjYdHaVhbbf9v8qqaqFr0xc9vqFj0dXdbba91qpepeI8k8fiI+fsY=rqGqVepae9pg0db9vqaiVgFr0xfr=xfr=xc9adbaqaaeGacaGaaiaabeqaaeqabiWaaaGcbaGaeeysaKKaeeOBa4Maee4yamMaeeyAaKMaeeizaqMaeeyzauMaeeOBa4Maee4yamMaeeyzauMaeeiiaaIaee4Ba8MaeeOzayMaeeiiaaIaeeyta0KaeeiraqKaeeOuaiLaeeyla0IaeeivaqLaeeOqaiKaeeiiaaIaeeiCaaNaeeyzauMaeeOCaiNaeeiiaaIaeeymaeJaeeimaaJaeeimaaJaeeilaWIaeeimaaJaeeimaaJaeeimaaJaeeiiaaIaee4Ba8MaeeOzayMaeeiiaaIaeeiDaqNaeeiAaGMaeeyzauMaeeiiaaIaeeiCaaNaee4Ba8MaeeiCaaNaeeyDauNaeeiBaWMaeeyyaeMaeeiDaqNaeeyAaKMaee4Ba8MaeeOBa4Maeyypa0tcfa4aaSaaaeaacqqGqbaucqqGYbGCcqqGVbWBcqqGWbaCcqqGVbWBcqqGYbGCcqqG0baDcqqGPbqAcqqGVbWBcqqGUbGBcqqGGaaicqqGubavcqqGcbGqcqqGGaaicqqGJbWycqqGHbqycqqGZbWCcqqGLbqzcqqGZbWCcqqGGaaicqqGnbqtcqqGebarcqqGsbGuaeaacqaIXaqmcqaIWaamcqaIWaamaaGccqGHxdaTcqqGjbqscqqGUbGBcqqGJbWycqqGPbqAcqqGKbazcqqGLbqzcqqGUbGBcqqGJbWycqqGLbqzcqqGGaaicqqGVbWBcqqGMbGzcqqGGaaicqqGubavcqqGcbGqcqqGGaaicqqGWbaCcqqGLbqzcqqGYbGCcqqGGaaicqaIXaqmcqaIWaamcqaIWaamcqGGSaalcqaIWaamcqaIWaamcqaIWaamaaa@A700@

Data from 97 countries or geographical settings was compiled and ranked according to the estimated incidence of transmitted MDR-TB within the population. Surveys undertaken in Andorra, Cambodia, Iceland, Luxembourg, Malta, New Zealand, Oman, Slovenia and Switzerland reported no MDR-TB amongst previously untreated cases. In just over half (49/97) of the geographical settings analysed, the estimated incidence of transmitted MDR-TB was less than 1 case per 100,000 of the population. Fourteen geographical settings had an estimated incidence of between one and three MDR-TB cases per 100,000: Casablanca (Morocco), Guangdon Province (China), Zhejian Province (China), Guinea, Israel, Honduras, Orel Oblast (Russian Federation), Republic of Korea, Nepal, Nicaragua, Iran, Thailand, Uganda (partial survey) and Sierra Leone. The 25 settings with an estimated incidence greater than three per 100,000 are presented in Table 1 in descending order of estimated incidence. The incidence of MDR-TB as previously reported is presented for each setting with a ranking to reflect the comparative degree of resistance. Whereas Karakalpakstan has the highest estimated incidence of MDR-TB at 35.3 cases per 100,000 of the population, Kazakhstan with 14.2% has the highest proportion of new cases that are MDR-TB. The list includes Zambia, Mozambique, Botswana and all eight of the South African provinces for which data was available. Estimates of the number of MDR-TB cases were high for each three of the Chinese settings, reflecting the large population of this region.

**Table 1:** Multi-drug resistance in new cases of tuberculosis with an estimated incidence greater than 3 per 100,000 of the population ranked in order of descending incidence.

**Country/Setting**	**Date of survey**	**Population surveyed**	**Estimated incidence TB per 100,000**	**Estimated number of MDR-TB cases**	**Estimated incidence MDR-TB per 100,000**	**% TB cases with MDR**	**Ranking by % MDR**
Karakalpakstan (Uzbekistan)	2001–2002	1,527,009	267.4	539	35.30	13.2%	3
Kazakhstan	2001	14,831,400	155.7	3,279	22.11	14.2%	1
Mpumalanga Province (SA)	2001–2002	3,111,069	578	468	15.03	2.6%	17
Kwazulu-Natal Province (SA)	2001–2002	9,146,297	827	1,286	14.06	1.7%	25
Tomsk Oblast (Russ. Fed.)	2002	941,278	93^1^	120	12.74	13.7%	2
North Arcot District (India)	1999	5,664,823	400	634	11.20	2.8%	16
North West Province (SA)	2002–2002	3,625,924	486	388	10.69	2.2%	20
Limpopo Province (SA)	2001–2002	5,683,605	443	604	10.63	2.4%	19
Free State Province (SA)	2001–2002	2,834,519	530	270	9.54	1.8%	24
Gauteng Province (SA)	2001–2002	8,020,408	670	752	9.38	1.4%	27
Hubei Province (China)	1999	59,165,000	440	5,467	9.24	2.1%	21
Mozambique	1998–1999	16,916,638	254	1,504	8.89	3.5%	13
Eastern Cape Province (SA)	2001–2002	7,001,260	875	613	8.75	1.0%	31
Zambia	2000	10,205,000^2^	475	891	8.55	1.8%	24
Western Cape Province (SA)	2001–2002	4,255,743	932	357	8.39	0.9%	32
Liaoning Province (China)	1999	40,900,000	80	3,403	8.32	10.4%	4
Peru	1999	25,232,226	265	2,006	7.95	3.0%	14
Latvia	2000	237,300	82^1^	181	7.63	9.3%	6
Lithuania	2002	3,487,000	74.7	245	7.02	9.4%	5
Estonia	2000	1,369,515	55.5^1^	93	6.77	12.2%	3
Botswana	2002	1,680,863	620	83	4.96	0.8%	33
Ivanovo Oblast (Russian Fed.)	1998	1,271,100	52	59	4.68	9.0%	7
Dashoguz Velayat (Turkmenistan)	2001–2002	1,141,900	92.9	40	3.53	3.8%	12
Raichur District (India)	1999	1,783,822	127	57	3.18	2.5%	18
Henan Province (China)	2001	94,350,000	38.5^1^	2,833	3.00	7.8%	8

^1 ^"Notification all cases" estimated incidence rate not available. ^2 ^Population from US Census Bureau's International Data Base. SA = Republic of South Africa.

While is it reassuring that in the majority of settings surveyed the estimated incidence of MDR-TB in new cases was found to be low, the incidence in some settings is alarming. A number of sub-Saharan countries that were previously considered to have low burdens of drug resistance were found to have amongst the highest estimated incidence of transmitted MDR-TB in the world. KwaZulu-Natal Province of South Africa which recently reported an outbreak of XDR-TB [15] had reported a low proportion of cases that were MDR (1.7%) and was previously ranked 25^th ^in the list of high prevalence MDR-TB countries. However, our analysis suggests that with an estimated 14 cases per 100,000 of the population it has the 4^th ^highest incidence of transmitted MDR-TB so far reported. We suggest that while data on the proportion of cases that are resistant to anti-tuberculosis drugs offers valuable guidance on the effectiveness of first line treatment programs it does not reflect the burden of drug resistant disease on a community, or the scale of intervention required to interrupt transmission. If high incidence of transmitted MDR-TB is a risk factor for the emergence and spread of untreatable XDR disease then interventions to control MDR-TB are urgently needed in all settings with elevated incidences, including those of sub-Saharan Africa.

To obtain a complete picture of the incidence or prevalence of MDR-TB in a population would require the inclusion of MDR-TB that has emerged in previously treated cases. Unfortunately sampling strategies for previously treated cases varied in the reported studies, and in some cases was not representative of the general population. In the absence of a full data set only data on new cases were selected for analysis. The estimated incidence of MDR-TB in new cases may therefore be considered an underestimate of the total incidence of MDR-TB. It is acknowledged that the global picture of drug resistance is far from complete as many countries in which TB is endemic have yet to be surveyed, or were surveyed some years ago [16]. Clearly, expanded surveillance activities are urgently needed to allow a fuller assessment of the burden of MDR-TB across the world.

We propose that when assessing the impact of drug resistance in a geographical setting it would be appropriate to consider its incidence in terms of the population. We recommend that surveillance activities are extended to permit estimation of the incidence and prevalence of drug resistant tuberculosis in the population.

Preventing the emergence of XDR-TB is a primary concern worldwide. The ongoing outbreak of XDR-TB and high deaths rates witnessed in South Africa has prompted calls for increased resources. WHO has estimated that an extra US$ 400 million will be needed to fund global MDR-TB and XDR-TB control activities in 2007 [17]. Unfortunately the appeal issued by WHO for additional funds has so far generated little response [18]. We call on the international donor community to recognise the threat of drug resistant tuberculosis to sub Saharan Africa and other regions of the world and to mobilise the necessary resources for its control.

## Summary

• The emergence of drug resistance is a serious threat to global efforts to control tuberculosis.

• The practice of expressing the prevalence of MDR-TB in a geographic setting as the proportion of cases found resistant does not adequately reflect the burden of MDR-TB within the community or the level of transmission of drug resistant disease.

• We recommend that global surveillance activities are expanded to include population based statistics on the incidence and prevalence of MDR-TB.

• Re-appraisal of global drug resistance data suggests that the problem of drug resistant tuberculosis in sub-Saharan Africa is more critical than previously perceived.

## Abbreviations

MDR-TB: Multidrug-resistant tuberculosis; WHO: World Health Organisation; XDR-TB Extensively drug-resistant tuberculosis.

## Competing interests

The author(s) declare that they have no competing interests.

## Authors' contributions

EZ contributed to study design, data analysis and drafting the article.

RM contributed to study conception, interpretation of data and drafting the article.

Both authors revised and approved the final version for publication.

## Pre-publication history

The pre-publication history for this paper can be accessed here:


